# Experimental Study on Time-Frequency Analysis of Vibration Signals from an Active De-Icing Exciter on Transmission Lines

**DOI:** 10.3390/s26134128

**Published:** 2026-06-30

**Authors:** Dongwang Fan, Bin Zhao, Mengxuan Li, Hao Wang, Lei Ding

**Affiliations:** 1 College of Mechanical Engineering, Tianjin University of Science and Technology, Tianjin 300457, China; fandw1117@mail.tust.edu.cn (D.F.); 15122435862@163.com (L.D.); 2 State Grid Electric Power Engineering Research Institute Co., Ltd., Beijing 100069, China; 17534921388@163.com (B.Z.); 15030065610@163.com (M.L.)

**Keywords:** overhead transmission line, active de-icing, time-frequency analysis, transient impact, low-pass filtering effect, nonlinear coupling

## Abstract

In traditional mechanical de-icing technologies, the time-frequency evolution and spatial propagation mechanisms of transient high-frequency impact signals in flexible transmission lines remain unclear. To address this issue, transient impact responses were experimentally investigated using a full-scale transmission line model. An active de-icing exciter, featuring controllable impact energy and the potential for sustained online operation, was independently developed. High-frequency transient acceleration signals were acquired at multiple measurement points on a 20 m single-span line. The spatial distribution and time-frequency attenuation characteristics of the impact energy were quantitatively evaluated by extracting high-order time-domain statistical features, including root mean square, kurtosis, and crest factor, together with frequency-domain analyses based on Fast Fourier Transform (FFT) and wavelet entropy. The results indicate that: (1) The exciter generated highly impulsive transient responses, with a kurtosis up to 795.3 and a crest factor approaching 40. This suggests a strong local concentration of impact energy at the excitation source, which provides a dynamic basis for analyzing potential localized stress concentration and dynamic responses of the conductor system. (2) The transmission line structure exhibited a significant low-pass filtering effect on transient high-frequency shock waves. As the shock wave propagated towards the distal end, its high-frequency components above 30 Hz were substantially attenuated, likely due to internal dry friction within the stranded conductor. Consequently, the dominant frequency decreased to a low-frequency macroscopic sway of approximately 12 Hz, indicating a reduced risk of transmitting high-frequency shock loads to distal fittings and towers. (3) Under geometric nonlinear coupling, the vertical impact energy was partially transferred to the longitudinal and lateral directions during propagation, leading to sustained out-of-plane swaying. This study reveals the signal evolution characteristics of transient impacts in overhead transmission lines and provides experimental evidence for optimizing excitation parameters and assessing the engineering safety of active impact de-icing technologies.

## 1. Introduction

Overhead transmission lines are core components of power transmission systems, and their operational safety is directly related to the stability and reliability of power supply. However, with the frequent occurrence of extreme weather events, large-span flexible transmission lines are highly susceptible to severe icing disasters in winter [1]. Continuous icing not only significantly increases the static mechanical load on conductors, but may also induce strong transient vibration waves during ice shedding or external mechanical impacts. The resulting dynamic stresses may exceed the safety margins of fittings, leading to the breakage of insulator strings or, in severe cases, tower collapse and conductor failure [2,3]. Therefore, clarifying the evolution characteristics of impact-induced vibration signals in transmission lines and developing efficient active de-icing technologies are essential for improving the operational safety of modern power grids [4].

Recent studies on transmission line de-icing have mainly focused on two aspects: improving de-icing technologies and characterizing the dynamic responses of conductors under external excitation. Conventional passive anti-icing coatings and thermal ice-melting methods are often limited by coating degradation, high energy consumption, and restricted applicability under online operating conditions [5]. By contrast, mechanical de-icing methods can generate dynamic responses in conductors through external impacts or forced vibration, thereby promoting ice fracture and weakening the ice–conductor adhesion interface [6,7]. Because the effectiveness and safety of mechanical de-icing largely depend on how the injected vibration energy propagates and attenuates along the conductor, accurate characterization of impact-induced vibration signals is essential. Accordingly, various signal-processing and time-frequency analysis methods have been introduced to identify the non-stationary dynamic characteristics of cable and conductor systems. Techniques such as Short-Time Fourier Transform (STFT), Variational Mode Decomposition (VMD), and Hilbert Huang Transform (HHT) have been used to extract time-varying frequencies and characteristic frequency bands associated with aeolian vibration, broken strands, structural faults, and transient excitation [8,9,10,11]. In addition, multi-point monitoring studies on transmission lines have shown that high-frequency acceleration responses may exhibit strong spatial variability under asymmetric excitation, highlighting the need to examine vibration propagation from both temporal and spatial perspectives [12]. Additionally, while modern data-driven methodologies such as capsule networks and algorithm unrolling networks [13,14,15] have been discussed for complex vibration interpretation, their application to transmission lines requires high-fidelity physical baseline datasets. This study focuses on providing such baseline experimental measurements for stranded conductors, ensuring an empirical foundation for future physical-informed modeling.

However, several limitations remain in current studies on mechanical de-icing and vibration signal analysis. First, many numerical models have difficulty accurately representing the dry-friction damping and filtering effects of multi-strand stranded conductors on high-frequency transient impact signals, which may lead to deviations between simulated and actual dynamic responses [16]. Second, existing physical experiments often rely on low-frequency continuous excitation devices, making it difficult to reproduce the high-energy and narrow-pulse transient characteristics associated with impact-based de-icing scenarios. More importantly, the time-frequency evolution and spatial attenuation characteristics of real transient impact signals in full-scale flexible transmission lines remain insufficiently understood [17]. In particular, the filtering behavior of high-frequency impact waves during propagation along stranded conductors, as well as the three-dimensional transfer and dissipation of transient vibration energy, has not yet been fully clarified. This lack of understanding limits the rational selection of excitation energy and the determination of installation spacing for active de-icing devices, making it challenging to balance de-icing effectiveness with the structural safety of transmission lines and associated fittings [18].

To address these limitations, this study aims to clarify the time-frequency evolution and spatial attenuation characteristics of impact-induced vibration responses in flexible transmission lines. A drop-hammer active de-icing exciter with controllable impact energy was independently developed, and transient acceleration responses were measured at multiple points on a full-scale 20 m single-span transmission line. By combining time-domain statistical indicators with frequency-domain and time-frequency analysis methods, including Fast Fourier Transform (FFT), spectral entropy, and wavelet entropy, this study quantifies the attenuation of high-frequency impact components and the directional redistribution of vibration energy during propagation. While the individual mathematical tools employed in this study (e.g., FFT, Kurtosis, and Wavelet Entropy) are well-established, their systematic integration provides a practical framework to evaluate the transient responses of flexible systems. By utilizing this multi-scale characterization, this work analyzes high-frequency transient shock propagation in stranded conductors, focusing on the structural low-pass filtering effect of inter-strand dry friction and the cross-axis energy redistribution under geometric coupling. These findings offer physical data to optimize active impact parameters and evaluate dynamic line safety. The specific objectives are to: (i) characterize the transient output features of the active de-icing exciter; (ii) evaluate the spatial attenuation of impact-induced vibration responses along the conductor; and (iii) identify the frequency-domain filtering and three-dimensional energy redistribution characteristics of the transmission line under impact excitation.

## 2. Experimental Set-Up and Test Protocol

### 2.1. Design and Working Principle of Active De-Icing Vibration Exciter

To address the difficulty of efficiently removing ice from overhead transmission lines, existing experiments typically simulate impacts by cutting ropes or employing manual striking. However, these methods suffer from critical flaws, including uncontrollable energy, random contact states, and poor repeatability. To overcome these limitations, an active de-icing exciter specifically designed for overhead transmission lines was independently developed in this study, and a single-span real transmission line testing platform was constructed at the experimental base.

**Figure 1:**
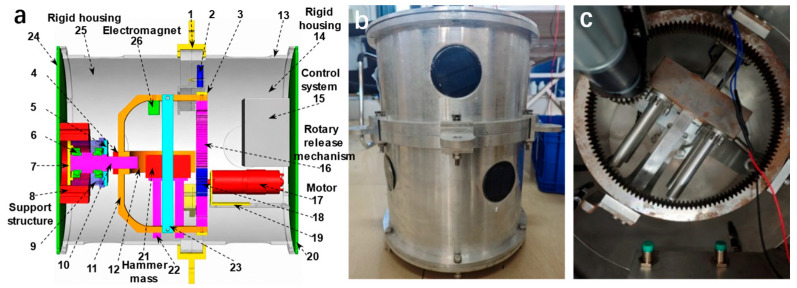
Configuration of the active de-icing exciter: (**a**) three-dimensional model; (**b**) prototype; and (**c**) internal structure.

The core design of this de-icing exciter aims to release high-energy transient pulses, thereby exciting intense local shear stresses within the conductor sufficient to destroy the ice adhesion. As shown in Figure 1a, the device mainly consists of the following core components: a motor (17) and a rotary release mechanism (16 and 18) that lift the drop hammer and precisely control its initial height to guarantee consistent potential energy for each drop; an electromagnet (26) acting as the key trigger actuator, which allows the drop hammer to free-fall with zero initial velocity upon power loss, effectively eliminating initial disturbances caused by traditional mechanical jamming; a 3.6 kg drop hammer mass (21) serving as the core high-energy excitation source; a rigid outer shell (14 and 25) and support structure (8) machined from high-strength aluminum alloy to ensure overall structural stiffness and lossless transmission of the bottom collision impact energy to the conductor; and a control system (15) for automated loading control and synchronized data acquisition triggering.

Its working principle is as follows: Before the experiment begins, the motor drives the rotary release mechanism to lift the drop hammer to the top vertex. When the de-icing command is triggered, the electromagnet is powered off, and the drop hammer falls freely, resulting in a vertical, hard collision with the rigid base at the bottom of the device. The extreme transient impact force generated by the collision couples directly to the transmission line, inducing high-frequency stress waves and large-amplitude nonlinear swaying within the conductor. As depicted in the physical photograph in Figure 1b,c, this design ensures high consistency in the contact position, contact angle, and initial energy of each impact, thereby guaranteeing the repeatability of the experimental data.

### 2.2. Technical Parameters and Impact Dynamics Estimation of Vibration Exciter

To ensure the reliability of the experiment and a standardized output of impact energy, the key technical parameters of the active de-icing exciter were strictly designed and calibrated. The specific parameters are detailed in Table 1. To guarantee that the input impact energy is controllable and repeatable, the drop hammer’s energy is physically secured by three parameters: a constant mass (3.6 kg), a constant drop height (150 mm), and an electromagnetic power-off release ensuring a zero initial velocity. This configuration guarantees that the initial energy remains consistent for each independent strike.

To provide a preliminary, first-order estimate of the baseline impact force magnitude, the average impact force was evaluated based on the classical impulse-momentum theorem. Given a drop height of H, and assuming an ideal state that ignores friction losses, the velocity of the drop hammer at the moment of contact is $v_{0}=\sqrt{2gH}$. The collision between the drop hammer and the high-strength aluminum alloy base is assumed to be an inelastic collision (with a restitution coefficient e set to 0.3). Additionally, the collision duration is extremely short (Δt is typically at the millisecond level). According to the impulse-momentum theorem, the generated average transient impact force F can be approximately expressed as:

(1) $$\bar{F}=\frac{m_{0}v_{0}\left(1+e\right)}{\Delta t}$$

while this simplified analytical calculation neglects contact compliance, structural elastic deformation of the colliding bodies, and non-linear energy loss mechanisms, it serves as a baseline order-of-magnitude approximation to verify that the system’s dynamic excitation capability reaches the kilonewton scale. Based on system parameters and pre-experimental calibration, this device can output an instantaneous peak impact force exceeding 8 kN, with an impact duration Δt of only about 1 ms. This “ultra-short duration, ultra-high energy” pulse characteristic generates high-amplitude transient dynamic features near the excitation source. Consequently, it serves as a baseline mechanical source for evaluating the dynamic response characteristics of the active impact excitation.

### 2.3. Transmission Line Experimental Platform and Multi-Point Monitoring Scheme

The experimental platform was established in a large indoor laboratory free from significant wind field interference. To avoid coupling interference from spacer dampers, dynamic testing was performed on a single-span bare conductor. The experimental span was set to L = 20 m. Both ends of the conductor were horizontally fixed by rigid anchorages without any height difference (constructed of heavy-duty thick-walled steel frames rigidly bolted to the laboratory’s concrete floor foundation to ensure that potential boundary micro-displacements remain on a negligible micrometer scale). The conductor used in the experiment was the commonly used A3/S3A-732/92 [19] aluminum conductor steel reinforced (ACSR), with its installation tension controlled at 10.739 MPa (corresponding to a tensile force of 8.856 kN, which was applied via a heavy-duty turnbuckle tensioner and precisely monitored in real-time using a high-precision S-type tensile load cell connected in-series at the anchorage terminal). The physical and geometric parameters of the conductor are detailed in Table 2.

To accurately capture the spatial transmission and attenuation characteristics of high-frequency impact energy along the conductor, a total of three triaxial piezoelectric high-frequency acceleration sensors (IEPE), designated as S1, S2, and S3, were deployed in the experiment. Excitation source measurement point (S2): The active de-icing exciter was securely clamped to the conductor at approximately one-third of the total length using high-strength bolts. The S2 sensor was mounted directly on the exciter to capture the initial injection characteristics of the de-icing energy. Near-end measurement point (S1): Located 5 m away from the origin, this point was used to monitor the attenuation laws of the shock wave in the near-field region. Far-end measurement point (S3): Located 15 m away from the origin, this point was used to evaluate the filtering effect and the safety of the tower attachment point as the shock wave propagates to the distal end.

**Figure 2:**
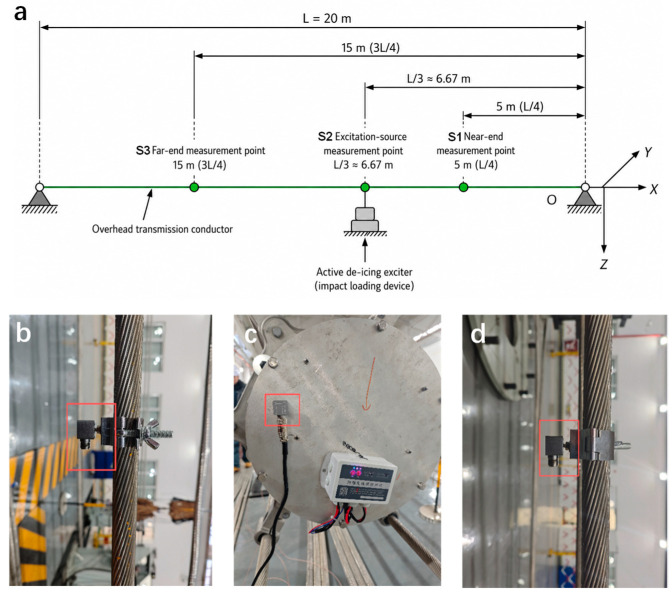
Experimental setup and field installation of acceleration sensors: (**a**) schematic diagram of the test platform; (**b**–**d**) installation details of sensors S1, S2, and S3, respectively.

A globally unified Cartesian coordinate system (X-Y-Z) was established for the experiment: the X-axis lies along the horizontal axial direction of the conductor, the Y-axis is perpendicular to the vertical plane containing the conductor (lateral direction), and the Z-axis points vertically downward (aligning with the main impact direction). The schematic diagram of sensor layout is shown in Figure 2a, and the specific installation position is shown in Figure 2b–d. See Table 3 for the corresponding relationship between sensor parameters and coordinate system.

The data acquisition terminal employed a NET-2412 series high-precision networked synchronous data acquisition card (DAQ), featuring a 24-bit Sigma-Delta (Σ−Δ) analog-to-digital converter (ADC) with an oversampling architecture. To precisely capture the high-frequency excitation signals with pulse widths of only milliseconds and to prevent frequency aliasing, the sampling frequency for all channels was uniformly set to 1 kHz. establishing a Nyquist folding frequency of 500 Hz. Physically, the transient impact force produced by the drop-hammer is represented as a half-sine pulse with a finite contact duration of approximately τ≈10 ms, which concentrates over 95% of the input energy within the low-frequency band below 1.5/τ=150 Hz. Due to the natural low-pass filtering of multi-strand ACSR conductors caused by inter-strand dry friction, the dynamic structural responses are restricted within this low-frequency band, ensuring that the 500 Hz limit provides a substantial 3.3-fold safety margin with no loss of critical dynamic information. With the sampling rate set to 1 kHz, the built-in digital decimation filter automatically scales and configures its anti-aliasing cutoff frequency to 450 Hz (0.45 $\times \;f_{s}$), which features a steep roll-off attenuation (attenuation > 100 dB near the Nyquist frequency of 500 Hz) to eliminate high-frequency aliasing.

To comprehensively decouple the inherent characteristics of the exciter body from the nonlinear coupling response of the conductor, the following two sets of typical experimental conditions were designed. Condition A (No-load baseline test): Before being mounted on the transmission line, the exciter was completely detached from the conductor, placed and secured on the solid concrete ground of the laboratory. It was then triggered independently. Its triaxial acceleration responses were recorded to extract the output energy level and the background modal interference of the device itself, guaranteeing that the measured signals represent only the inherent mechanical output of the exciter body without coupling effect from the flexible conductor. Condition B (On-line test): The exciter was installed at measurement point S2 on the conductor. A single drop-hammer impact was applied, while the transient triaxial accelerations at three spatial measurement points (S1, S2, and S3) were synchronously recorded at high frequency to investigate the spatiotemporal evolution laws of the shock wave. To reduce the randomness during the dynamic impact process of the active de-icing exciter and ensure the reliability of the experimental data, the tests under identical operating conditions were repeated five times. A multi-axial statistical dispersion and graphical confidence interval analysis of these repeated trials are detailed.

## 3. Analysis and Discussion of Experimental Results

To verify the reliability and stability of the dynamic measurements before diving into detailed waveform analysis, a comprehensive statistical evaluation of the key phase-invariant scalar indicators (Peak Acceleration and Dominant Frequency) across the five repeated trials (N = 5) was performed for all three axes (X, Y, and Z). The statistical results-including the Sample Mean ($\bar{x}$), Standard Deviation (SD), and the Coefficient of Variation (CV%)—are summarized in Table 4. This analysis focuses on the high-Signal-to-Noise Ratio (SNR) data collected from the No-Load Device Body (characterizing the exciter’s output consistency) and the On-Line S2 Point (characterizing the conductor’s dynamic response near the source).

**Figure 3:**
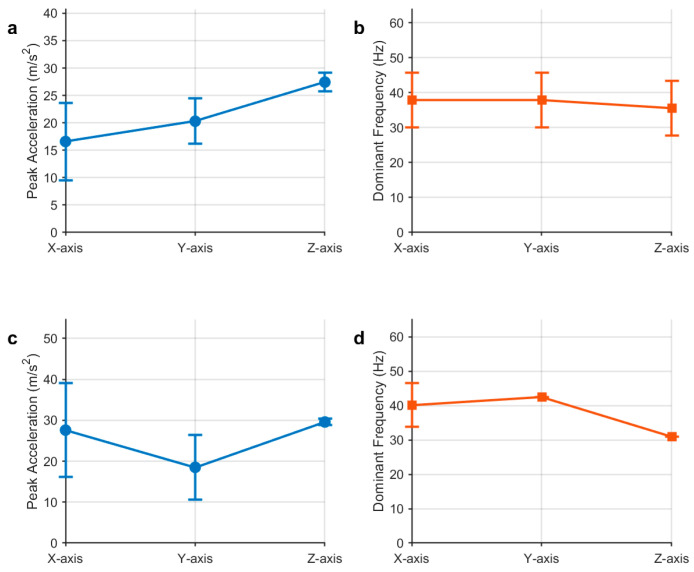
Triaxial peak accelerations and dominant frequencies distributions and their corresponding 95% confidence intervals (indicated by error bars) across 5 repeated trials under: (**a**) No-load Condition (peak acceleration), and (**b**) No-load Condition (dominant frequency), and (**c**) On-line Condition (peak acceleration), and (**d**) On-line Condition (dominant frequency).

Furthermore, to visually evaluate the data dispersion and statistical stability, the 95% confidence intervals (CIs) of the triaxial peak accelerations and dominant frequencies were calculated based on Student’s t-distribution (t = 0.025, 4 = 2.776 for N = 5) and plotted as Figure 3, respectively. Crucially, to ensure statistical reliability, No-Load Trial 1 and On-Line Trial 3 were selected as the typical representative runs for the detailed comparative waveforms and 3D frequency spectra in the subsequent sections (Section 3.1, Section 3.2, Section 3.3 and Section 3.4). These typical trials were chosen because their peak accelerations and frequency characteristics closely align with the statistical average of the five runs, making them ideal to illustrate the general physical wave propagation and damping behaviors of the system.

The statistical data in Table 4 and the graphical confidence intervals in Figure 3 show the dynamic response characteristics among the three axes. The vertical vibration direction (Z axis)—which is the direct path of the drop-hammer’s impact—displays high repeatability. Under the On-line S2 condition, the Z axis peak acceleration has a CV of 2.10%, and its dominant frequency is consistent at 30.98 Hz, indicating that the initial energy input from the excitation mechanism is repeatable.

In contrast, the passive coupled directions (X axis and Y axis) exhibit higher data dispersion, with CV values of peak acceleration ranging from 16.45% to 34.42%. Physically, the X and Y axes are not directly excited; their dynamic vibrations are driven by the vertical impact through the three-dimensional geometric coupling and wave mode conversion in the flexible conductor. Because the stranded conductor consists of multiple layers of aluminum wires, micro-slips and dry friction inside the multi-strand structure can introduce variations under dynamic tension fluctuations. Similarly, the minor shifting of dominant frequencies between the closely coupled structural modal frequencies (~31 Hz and ~42 Hz) reflects discrete FFT bin selection under modal energy redistribution. These multi-axial characteristics align with the physical behavior of multi-strand flexible conductors under dynamic impacts, providing a baseline reference for subsequent discussions.

### 3.1. Time-Domain Feature Analysis of the Exciter’s Output Signal

To accurately evaluate the mechanical output performance, impact purity, and energy distribution characteristics of the independently developed active de-icing exciter before it is mounted on a real transmission line, a no-load drop-hammer calibration experiment was first conducted. Using a high-frequency data acquisition system, the transient acceleration responses of the exciter body in three orthogonal directions—X (longitudinal), Y (lateral), and Z (vertical)—were synchronously recorded.

**Figure 4:**
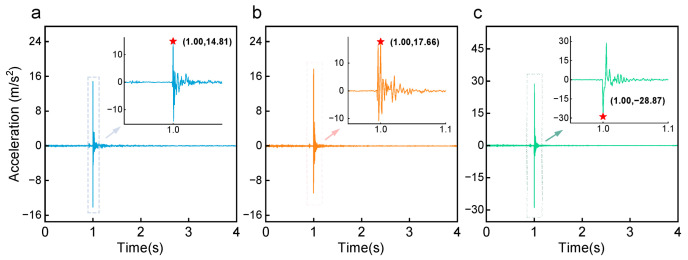
Time domain diagram of three-dimensional acceleration under no-load state of vibration exciter: (**a**) X-axis time domain; (**b**) Y-axis time domain and (**c**) Z-axis time domain, ★ indicates the peak of acceleration.

Figure 4a–c display the time-domain waveforms of the triaxial acceleration in the X, Y, and Z directions, respectively, under a single no-load trigger. Combined with the local enlarged views, it can be intuitively observed that at the instant the drop hammer strikes the base, an extremely sharp acceleration pulse is generated in the main impact direction (Z axis), with a negative peak instantaneously reaching −28.87 m/s^2^. In contrast, due to the rigid constraint and guiding effect of the mechanical structure, the parasitic vibration peaks in the X and Y axes are only 14.81 m/s^2^ and 17.66 m/s^2^, respectively. Furthermore, the duration of the main pulse is extremely short (the pulse width is only about 10 ms). This time-domain morphology of “high amplitude, narrow pulse width” highly approximates an ideal Dirac delta function excitation. These intuitive time-domain features indicate that the mechanical release mechanism not only possesses excellent directional stability (with energy highly concentrated along the Z axis) but also exhibits an extremely explosive energy release.

However, for such highly non-stationary transient impact signals, relying solely on intuitive observation of time-domain waveforms is insufficient to comprehensively reveal their dynamics and energy allocation laws. Therefore, this paper introduces dimensionless statistical feature indicators to conduct a deep quantitative analysis of the time-domain properties of the impact signal. For a discrete acceleration sequence $x_{i}$ of length N, the following four most representative higher-order statistical and amplitude-domain features were selected for calculation:

Root Mean Square (RMS): Reflects the effective energy and average power level of the vibration signal over the entire analysis time window.

(2) $$X_{rms}=\sqrt{\frac{1}{N}\sum_{i=1}^{N}x_{i}^{2}}$$

Kurtosis (K): As the fourth-order central moment of the signal, kurtosis is extremely sensitive to impact components in the signal. It reflects the degree to which the vibration signal deviates from a normal distribution; a larger value indicates a more extreme and sharper impact.

(3) $$K=\frac{\frac{1}{N}\sum_{i=1}^{N}{\left(x_{i}-\bar{x}\right)}^{4}}{X_{rms}^{4}}$$

Crest Factor ($C_{f}$): Defined as the ratio of the signal’s peak value to its effective value (RMS), it is used to measure the prominence of the transient impact peak against the overall background energy.

(4) $$C_{f}=\frac{X_{max}}{X_{rms}}$$

Impulse Factor ($I_{f}$): The ratio of the peak value to the absolute mean value of the signal, which is a crucial indicator for identifying abrupt mechanical behaviors in structural health monitoring.

(5) $$I_{f}=\frac{X_{max}}{\frac{1}{N}\sum_{i=1}^{N}|x_{i}|}$$

Based on the above formulas, the original triaxial acceleration signals were calculated, and the extracted detailed statistical feature indicators are shown in Table 5.

Combined with the quantitative data in Table 5, the following in-depth dynamics conclusions can be drawn: First, from the perspective of absolute energy levels, both the RMS (0.733) and Variance (0.536) of the Z axis are significantly higher than those of the X and Y axes. This also confirms from a statistical standpoint that, although minor lateral and longitudinal energy leakage exists in three-dimensional space, the vast majority of the drop hammer’s kinetic energy is efficiently converted into dynamic excitation energy along the Z axis. More importantly, Table 5 reveals the extreme transient destructive potential of the impact source. In classical mechanical vibration theory, the Kurtosis of a standard Gaussian stationary random vibration is a constant value of 3. However, the Kurtosis of the Z axis response of this exciter reaches an astonishing 1213.75, while the Kurtosis values for the X and Y axes also reach 867.9 and 694.3, respectively. In impact dynamics, such a massive fourth-order moment typically only occurs during high-energy projectile penetration, brittle fracture, or severe hard collisions between metal bodies. This indicates that our exciter generates an “explosive” mechanical impact resulting from energy being extremely compressed and released within milliseconds, rather than a progressive harmonic vibration.

Furthermore, the Crest Factor of 39.38 and Impulse Factor of 273.27 on the Z axis further corroborate this point. A Crest Factor approaching 40 implies that its instantaneous destructive force is 40 times its average background energy. This characteristic is of decisive guiding significance for the application of active de-icing on overhead transmission lines. Icing layers are typically brittle materials, and their failure mechanism mainly depends on whether the local transient stress exceeds their shear/tensile strength limit, rather than continuous low-amplitude fatigue. The transient strong shock wave output by this exciter, characterized by an extremely high Kurtosis and Crest Factor, is highly conducive to inducing localized energy concentration near the exciter without triggering massive global low-frequency oscillations of the span. While the actual interfacial de-icing process involves complex multi-physics coupling, the high-impulse shock wave (with Kurtosis up to 1213.75 and Crest Factor near 40) generated by this active exciter provides a dynamic foundation for mechanical interface excitation and localized structural deformation studies.

### 3.2. Frequency-Domain Feature Analysis of the Exciter’s Output Signal

**Figure 5:**
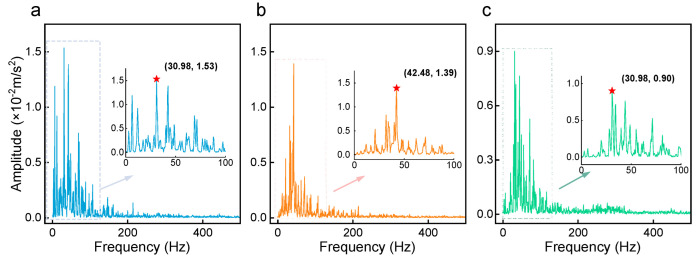
Frequency spectrum of triaxial acceleration of the exciter under no-load condition: (**a**) X-axis frequency spectrum; (**b**) Y-axis frequency spectrum and (**c**) Z-axis frequency spectrum, ★ indicates the peak of frequency.

After experiencing the instantaneous strong impact, the exciter body structure will undergo a period of free-decay vibration. The primary purpose of extracting the frequency-domain vibration characteristics of the exciter itself is to identify the specific vibration frequencies it generates. This provides fundamental reference data for the subsequent on-line experiments on real transmission lines, facilitating the identification and elimination of the exciter’s inherent frequency interference amidst complex coupled vibrations. To achieve this, a free-vibration segment from 1.5 s to 3.5 s in the time-domain signal was intercepted. To effectively suppress spectral leakage, a Hanning window was applied to the truncated signal before performing a Fast Fourier Transform (FFT). The obtained triaxial frequency spectra are shown in Figure 5.

To quantitatively measure the distribution characteristics of the signal frequency bands, this paper introduces the Spectral Centroid (SC), which characterizes the “center of mass” of the signal’s frequency band, and the Spectral Entropy (SE), which measures the complexity of the energy distribution. Their calculation formulas are as follows:

Spectral Centroid (SC): Reflects the “center of mass” of the signal’s spectral energy. A larger value indicates a higher proportion of high-frequency components contained in the signal.

(6) $$SC=\frac{\sum_{k=1}^{M}f_{k}\cdot A_{k}}{\sum_{k=1}^{M}A_{k}}$$

where $f_{k}$ is the frequency value of the k-th spectral line, $A_{k}$ is the corresponding amplitude, and M is the total number of spectral lines.

Spectral Entropy (SE): Based on information entropy theory, it reflects the degree of concentration of the system’s vibration energy across different frequency components. If the energy is highly concentrated at a single frequency, the Spectral Entropy approaches 0; conversely, if the energy is uniformly distributed across the frequency band, the Spectral Entropy is larger.

(7) $$SE=-\sum_{k=1}^{M}P_{k}\ln P_{k},\;P_{k}=\frac{A_{k}}{\sum_{k=1}^{M}A_{k}}$$

Combining the aforementioned frequency-domain indicators with Wavelet Energy features, the quantitative calculation results for the triaxial free-vibration signals are shown in Table 6.

From the data in Figure 5 and Table 6, it can be observed that during the free-vibration decay phase, the residual dominant frequency of the exciter in the X and Z axes is approximately 30.98 Hz, while the dominant frequency in the Y axis is approximately 42.47 Hz. Once these inherent frequencies are clarified, their interference can be effectively excluded in the subsequent frequency-domain analysis of the real transmission line. Analyzing the Wavelet Energy in Table 6 reveals that the residual energy of the Z axis during the free-vibration phase (6.02) is actually the lowest among the three axes. This indicates that the structure possesses excellent rigidity in the Z axis direction and does not produce severe low-frequency swaying after the impact. However, the Spectral Centroid of the Z axis (SC = 129.33 Hz) is significantly higher than those of the X and Y axes, indicating that its residual vibration is mainly composed of minor high-frequency aftershocks triggered by the high-frequency collision of structural components.

In summary, the no-load experiment fully quantifies the high-energy output characteristics of the active de-icing exciter and its own natural frequencies. This establishes a reliable physical foundation for subsequently exploring the dynamic response laws of overhead transmission lines under impact loads.

### 3.3. Time-Domain Response Analysis of the Transmission Conductor Under Single Excitation

**Figure 6:**
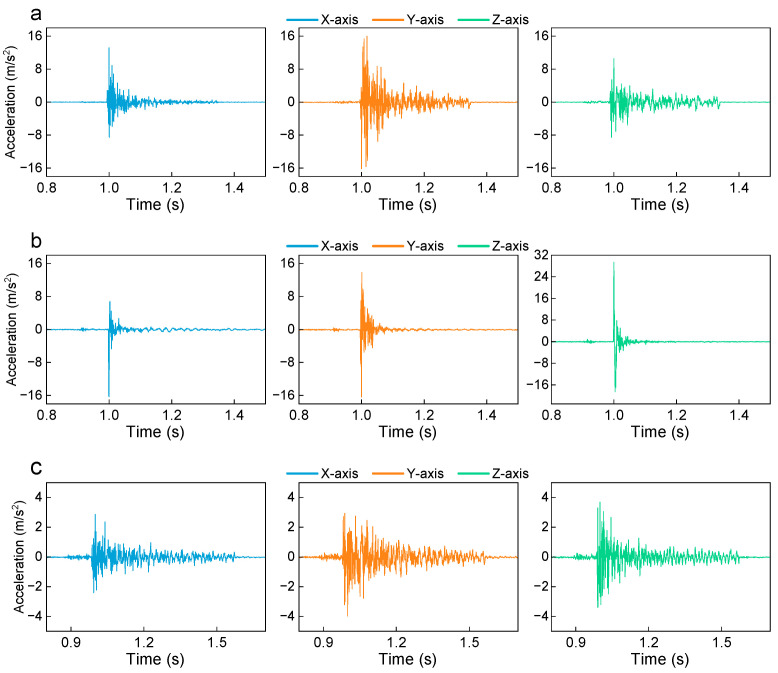
Time-domain triaxial acceleration charts of different measurement points under single impact: (**a**) S1 time domain; (**b**) S2 time domain and (**c**) S3 time domain.

After grasping the output baseline of the impact exciter, the exciter was officially mounted on the single-span real overhead transmission line platform for practical impact testing. According to the experimental setup, the exciter (excitation source) was installed at measurement point S2, approximately at L/3 of the conductor. To accurately capture the propagation and attenuation characteristics of the shock wave along both sides of the flexible conductor, measurement point S1 was located 5 m away from the origin, and the far-end measurement point S3 was located 15 m away. The transient triaxial acceleration response curves of these three measurement points during the forced vibration phase (approximately 0.8 s to 1.5 s) were extracted, as shown in Figure 6.

**Figure 7:**
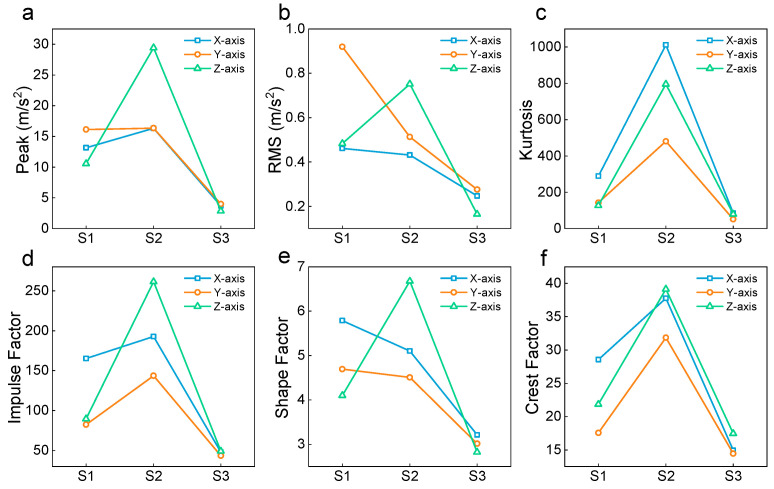
Time-domain feature indicators of three axes at different measurement points under single impact: (**a**) Peak; (**b**) RMS; (**c**) Kurtosis; (**d**) Impulse Factor; (**e**) Shape Factor and (**f**) Crest Factor.

Observing Figure 6, it can be seen that when the exciter applies a single Z axis drop-hammer impact at point S2, intense forced vibrations are rapidly excited across the entire transmission line in three-dimensional space. As the excitation source, measurement point S2 generates an extremely sharp, high-amplitude pulse along the Z axis. However, as the distance increases, the waveform amplitudes at the near-end S1 (5 m) and the far-end S3 (15 m) decrease significantly, and the originally sharp high-frequency burrs gradually smooth out. This intuitively demonstrates the spatial dissipation and filtering phenomena of shock waves within flexible media. To quantitatively analyze the underlying dynamics behind this phenomenon, a complete set of time-domain statistical features comprising over ten indicators was comprehensively extracted using signal processing algorithms. However, considering the targeted nature of the physical analysis, only six core key indicators (Peak, RMS, Kurtosis, Impulse Factor, Shape Factor, and Crest Factor) that most intuitively reflect the impact intensity and energy distribution were selected for in-depth discussion. The evolutionary line charts of these six indicators along the spatial positions of the conductor (S1-S2-S3) are shown in Figure 7.

Combining the evolutionary trends in Figure 7, the following core conclusions regarding the spatial transmission mechanism of impact energy along the transmission line can be drawn:

As clearly seen from Figure 7a,c,d,f, the data presents a highly typical “inverted-V” spatial distribution, meaning that all transient impact indicators at the excitation source (S2) reach their absolute maximums for the entire line. Taking the main impact Z axis as an example, the peak acceleration at S2 reaches 29.40 m/s^2^, its Kurtosis is as high as 795.3, and the Crest Factor approaches 40. These quantitative results perfectly corroborate the core mechanical logic of active impact de-icing: the exciter can instantaneously inject extremely high-density transient energy into the local area where it is installed. Such ultra-high Kurtosis and Crest Factor signify an extreme concentration of energy. These parameters indicate that the dynamic impact energy is highly localized near the excitation source, which is favorable for localized mechanical studies while minimizing the risk of transmitting severe dynamic shock loads to distal fittings.

As the shock wave propagates towards both sides, the high-frequency transient energy dissipates rapidly. The Peak, Kurtosis, and Crest Factor at the near-end S1 and far-end S3 measurement points all exhibit a precipitous drop. Particularly at the far-end S3 point, the Z axis peak value diminishes to merely 2.87 m/s^2^, and the Kurtosis drops to around 79. This completely deviates from the realm of destructive “hard impacts” and tends towards conventional, mild vibrations. This fully proves that the dry friction damping between the internal strands of the multi-strand overhead transmission line exerts an exceptionally strong attenuation effect on high-frequency shock waves. From an engineering application perspective, this natural “attenuation barrier” is highly advantageous. It implies that we can safely increase the single striking energy of the de-icing exciter without worrying that severe shock waves will propagate to the distal ends and damage critical fittings such as insulator strings and towers.

The most important discovery within this dataset is reflected in the RMS line chart (Figure 7b), which represents the effective energy. Although the main impact peak on the Z axis is highest at S2, the RMS value on the Y axis (lateral direction) at measurement point S1 surprisingly surpasses all other directions, reaching the line’s overall maximum of 0.919 m/s^2^. This anomalous phenomenon reveals the s “geometric nonlinear spatial coupling” of flexible cable networks under asymmetric transient loads. Specifically, with sensor cross-axis sensitivity below 3%, this multi-axial coupling represents a physical phenomenon rather than measurement artifacts. Mechanically, under rigid boundaries, the vertical impact induces transient tension fluctuations that act as parametric excitation, coupling vertical modes with lateral and longitudinal sways. This indicates a combined mechanism of geometric non-linearities and rigid boundary constraints in transferring dynamic energy downstream. This results in a “violent vertical hammering” near the impact point, whereas areas a few meters away evolve into a “continuous, high-energy lateral swaying.” This spatial energy transfer mechanism provides valuable experimental evidence for optimizing the installation spacing of the exciters and evaluating the wind deviation stability of the transmission lines.

### 3.4. Frequency-Domain Response Analysis of the Transmission Conductor Under Single Excitation

**Figure 8:**
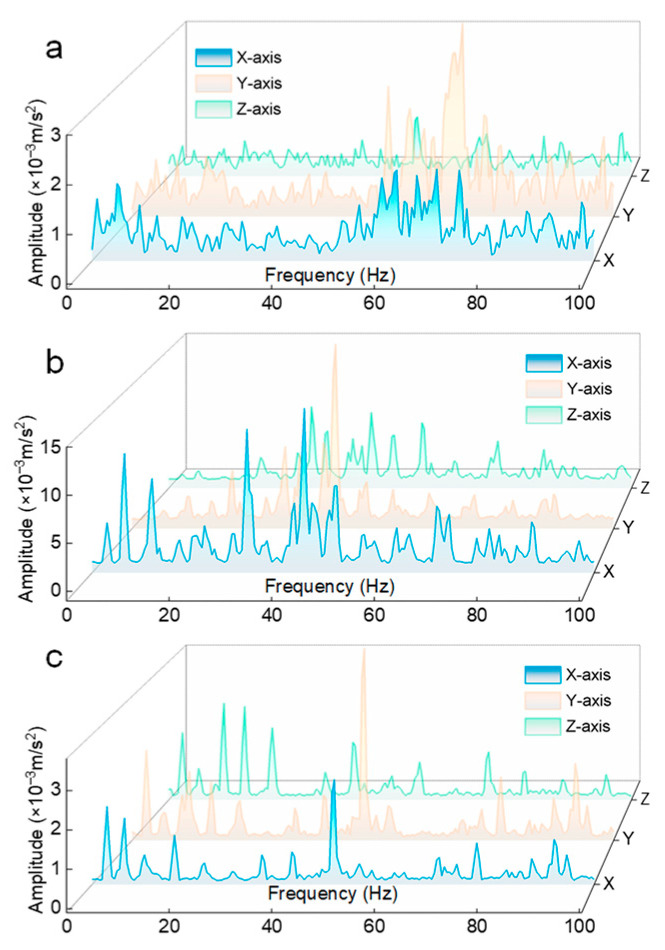
Frequency spectrum of three axes at different measurement points under single impact: (**a**) S1 Frequency spectrum; (**b**) S2 Frequency spectrum and (**c**) S3 Frequency spectrum.

**Figure 9:**
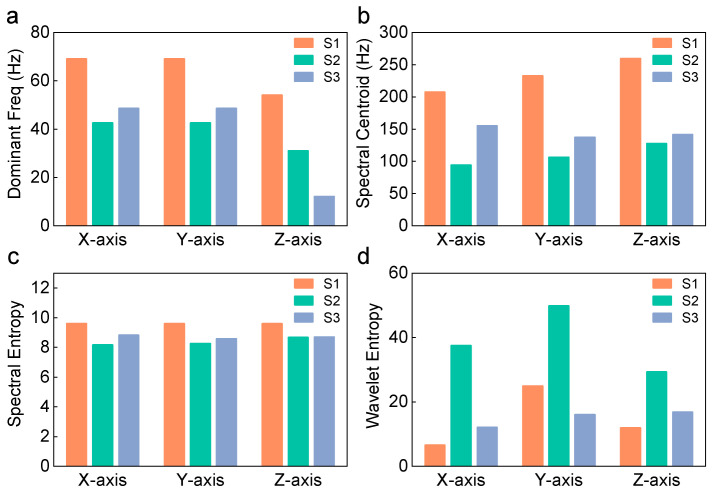
Frequency-domain feature indicators of three axes at different measurement points under single impact: (**a**) Dominant Freq; (**b**) Spectral Centroid; (**c**) Spectral Entropy and (**d**) Wavelet Entropy.

To further investigate the time-frequency evolution laws of the vibration energy induced by the impact load within the structure, free-vibration segments following the forced vibration phase were extracted from the signals at each measurement point. Similarly, a Hanning window was applied to the truncated signals prior to performing a Fast Fourier Transform (FFT). The resulting 3D frequency-domain waterfall plots for measurement points S1, S2, and S3 are shown in Figure 8. Concurrently, bar charts comparing four key frequency-domain feature indicators were generated, as shown in Figure 9.

Through comprehensive analysis of the frequency-domain waveforms and quantitative indicators, the core laws of energy distribution during the free vibration phase can be obtained. From the spectral amplitude distribution in Figure 8, it can be seen that the excitation source S2 (Figure 8b) retains the maximum overall vibration energy. Particularly, towering resonance peaks appear at specific frequencies on its X and Y axes, with maximum amplitudes exceeding 15 × 10 ^−3^ m/s^2^. In contrast, as the energy transmits outward to the near-end S1 (Figure 8a) and far-end S3 (Figure 8c), the peak amplitudes of the spectra on all axes attenuate significantly, generally maintaining a maximum of around 3 × 10 ^−3^ m/s^2^. This is consistent with the conclusion in the time-domain analysis, indicating that the high-energy amplitude used to shatter the ice is difficult to effectively transmit over long distances along the conductor.

Combined with the quantitative frequency-domain indicators in Figure 9, the filtering characteristics of the flexible transmission line body for different frequency waves, as well as their practical impact on de-icing engineering, can be further revealed.

Observing the dominant frequency bar chart (Figure 9a), it can be found that there are obvious spatial differences in the dominant frequency distribution across different measurement points. At the excitation source S2, the dominant frequency of the Z axis is about 31 Hz, while the X and Y axes are about 42 Hz. However, when the stress wave propagates to the far-end measurement point S3, located 15 m away, the dominant frequency of the Z axis experiences a sharp, precipitous drop, down to around 12 Hz. From a frequency-domain perspective, this data intuitively confirms that the stranded transmission line structure possesses a significant “low-pass filtering” characteristic. Because high-frequency stress waves have short wavelengths, they are highly prone to inducing relative sliding between internal strands during propagation along the line, and are thus largely dissipated by dry friction. This implies that the vibration reaching the distal transmission tower is reduced to merely low-frequency macroscopic swaying of approximately 12 Hz. Such gentle fluctuations are insufficient to damage distant insulator strings and other fittings, thereby serving as a natural protective mechanism.

The Spectral Centroid (Figure 9b) reflects the center of gravity of the vibration signal’s energy distribution. Across all three measurement points, the Spectral Centroids at the excitation source S2 are at the lowest level on all three axes (e.g., approximately 95 Hz for the X axis and 127 Hz for the Z axis). In contrast, at the more distant measurement points S1 and S3, the centroid frequencies all exhibit an upward shift (e.g., the Z axis centroid at S1 reaches as high as 260 Hz, and at S3 is about 141 Hz). From the perspective of the physical de-icing mechanism, at the impact origin S2, the enormous input of transient mechanical energy forcefully excites the large-amplitude fundamental mode of the local conductor, causing the energy to be highly concentrated in the low-frequency resonance band to produce destructive displacement. As the shock wave propagates towards the distal end, the low-frequency macroscopic displacement carrying large energy attenuates rapidly. Conversely, this causes the proportion of the residual weak high-frequency vibration components in the background relative to the total energy to increase, resulting in higher centroid values.

Wavelet Entropy and Spectral Entropy measure the complexity of the signal in terms of its time-frequency structure and frequency distribution, respectively. From Figure 9c,d, an extremely prominent phenomenon can be observed: the Wavelet Entropy at S2 is exceptionally massive, especially reaching approximately 50 on the Y axis and 37 on the X axis, while the corresponding Spectral Entropy remains at a relatively low level (around 8.1 to 8.2). This clearly indicates that the instantaneous impact at the excitation source generates a highly complex, multi-scale transient energy packet. This highly non-stationary, broadband excitation indicates a transient energy packet distributed across multiple scales near the source. As the wave propagates to S1 and S3, complex high-frequency impact components are filtered out by the wire body structure, and the multi-scale transient features fade. Consequently, the Wavelet Entropy at the distal ends drops significantly (e.g., all axes at the S3 measurement point decrease to around 12–17). This evolutionary process clearly depicts the physical transformation of the mechanical impact: transitioning from a violent, broadband, local transient excitation to the steady-state, multi-modal free vibration of the entire conductor.

## 4. Conclusions

This study experimentally investigated the time-frequency evolution and spatial propagation characteristics of impact-induced vibration responses in overhead transmission lines using an independently developed active de-icing exciter. The main conclusions are as follows:

1. A drop-hammer active de-icing exciter was developed and tested on a full-scale 20 m single-span transmission line platform. Multi-point triaxial high-frequency acceleration signals were acquired under transient impact excitation. Time-domain and time-frequency features, including kurtosis, crest factor, frequency spectra, spectral entropy, and wavelet entropy, were extracted to characterize the spatial attenuation and directional redistribution of impact-induced vibration energy in the flexible conductor system.
2. The exciter generated highly impulsive transient responses with a pulse width of approximately 10 ms. In the near-field region, the Crest Factor approached 40, and the Kurtosis reached 795.3, indicating a strong concentration of transient impact energy at the excitation source. Such highly impulsive responses are favorable for initiating localized vibration responses at the contact zone. Although the present study establishes the baseline wave propagation characteristics on a bare conductor, it provides the prerequisite physical reference (energy levels and attenuation boundaries) for subsequent validations under actual icing conditions, which are currently planned for our future work.
3. During propagation along the conductor, the impact-induced vibration response exhibited pronounced frequency-dependent attenuation and directional energy redistribution. High-frequency components were substantially attenuated, likely due to internal friction and structural damping within the stranded conductor, and the dominant frequency at the distal measurement point decreased from approximately 31 Hz to a low-frequency sway of about 12 Hz. This suggests a reduced risk of transmitting high-frequency shock loads to distal fittings and tower-side structures. Meanwhile, under geometric coupling of the flexible conductor, part of the vertical impact energy was transferred to the longitudinal and lateral directions, with the near-field lateral effective energy exceeding the vertical component. These findings provide experimental evidence for optimizing excitation parameters and installation spacing of active de-icing devices.

Future work should further consider finite element contact mechanics modeling to validate internal collision behaviors, real icing conditions, repeated impact excitation, and multi-span transmission line configurations to determine dynamic ice-breaking thresholds and improve the engineering applicability of active impact de-icing technologies.

## Figures and Tables

**Table 1:** Technical parameters of impact loading device (active de-icing exciter).

Total Mass of Installation/kg	Drop Weight Mass/kg	Effective Stroke/mm	External Diameter/mm	Release Response Time/ms	Release Synchronization Error/ms	Acceleration Peak Error/%
29.5	3.6	150	360	3	±1	4.8

**Table 2:** A3/S3A-732/92 conductor parameters.

**Young’s Modulus/** MPa	**Poisson’s Ratio**	Effective Cross-Sectional Area/${mm}^{2}$	External Diameter/mm	Linear Density/(kg/m)	Permissible Stress/MPa
6.7 × 10^4^	0.3	824.7	37.4	2.75	107.39

**Table 3:** Acceleration sensor layout and parameter index.

Station Number	S1	S2	S3
Arrangement Position	L/4 Places (On Conductor)	L/3 Places (Vibration Exciter Body)	3L/4 Places (On Conductor)
Correspondence Between Local and Global	Sensor x Axis→Global X Axis Sensor y Axis→Global Y Axis Sensor z Axis→Global Z Axis	Sensor x Axis→Global Z Axis Sensor y Axis→Global Y Axis Sensor z Axis→Global X Axis	Sensor x Axis→Global X Axis Sensor y Axis→Global Y Axis Sensor z Axis→Global Z Axis
Sensor Model	SAE30005	SAE30010	SAE30005
Range/g	50	100	50
Sensitivity/(mV/g)	X:102.0/Y:102.3/Z:103.5	X:49.1/Y:50.6/Z:50.8	X:102.0/Y:102.3/Z:103.5

**Table 4:** Statistical Repeatability Analysis of Triaxial Dynamic Indicators across 5 Repeated Trials.

Test Case	Dynamic Indicator	Trial 1	Trial 2	Trial 3	Trial 4	Trial 5	Mean	SD	CV (%)
No-Load (Device Body)	X-Peak Acc (m/s^2^)	14.81	17.87	21.87	7.63	20.77	16.59	5.71	34.42
	X-Dominant Freq (Hz)	30.98	42.47	42.47	30.98	42.47	37.87	6.29	16.62
	Y-Peak Acc (m/s^2^)	17.66	15.93	23.69	22.64	21.54	20.29	3.33	16.45
	Y-Dominant Freq (Hz)	42.47	42.47	30.98	30.98	42.47	37.87	6.29	16.61
	Z-Peak Acc (m/s^2^)	28.87	27.73	27.35	27.99	25.18	27.42	1.37	5.01
	Z-Dominant Freq (Hz)	30.98	30.98	42.47	42.47	30.98	35.58	6.29	17.69
Device Body(S2)	X-Peak Acc (m/s^2^)	24.29	41.29	16.31	25.32	30.80	27.60	9.24	33.46
	X-Dominant Freq (Hz)	30.98	42.47	42.47	42.47	42.47	40.17	5.14	12.79
	Y-Peak Acc (m/s^2^)	27.27	15.66	16.35	22.31	10.91	18.50	6.36	34.37
	Y-Dominant Freq (Hz)	42.47	42.47	42.47	42.47	42.47	42.47	0	0
	Z-Peak Acc (m/s^2^)	28.82	29.26	29.41	30.01	30.39	29.58	0.62	2.10
	Z-Dominant Freq (Hz)	30.98	30.98	30.98	30.98	30.98	30.98	0	0

**Table 5:** Time-domain signal feature values of triaxial acceleration of the exciter under no-load condition.

Indicator	X	Y	Z
Peak/(m/s^2^)	14.80	17.66	28.87
RMS(m/s^2^)	0.406	0.518	0.733
Peak2 (m/s^2^)	28.95	28.54	57.57
Std(m/s^2^)	0.40	0.51	0.73
Variance(m/s^2^)^2^	0.16	0.26	0.53
Kurtosis	867.95	694.3	1213.75
Impulse Factor	176.50	168.04	273.27
Shape Factor	4.84	4.93	6.93
Crest Factor	36.43	34.08	39.38
Clearance Factor	280.25	255.55	483.18

**Table 6:** Frequency-domain signal feature values of triaxial acceleration of the exciter under no-load condition.

Row	Dominant Freq/Hz	Spectral Centroid/Hz	Spectral Entropy	Wavelet Energy	Wavelet Entropy
X Axis	30.98	97.52	8.24	8.65	36.83
Y Axis	42.47	107.93	8.40	10.67	49.15
Z Axis	30.98	129.33	8.65	6.02	29.29

## Data Availability

Data will be made available on request.
